# Prediction Models for Railway Track Geometry Degradation Using Machine Learning Methods: A Review

**DOI:** 10.3390/s22197275

**Published:** 2022-09-26

**Authors:** Yingying Liao, Lei Han, Haoyu Wang, Hougui Zhang

**Affiliations:** 1 State Key Laboratory of Mechanical Behavior and System Safety of Traffic Engineering Structures, Shijiazhuang Tiedao University, Shijiazhuang 050043, China; 2 School of Civil Engineering, Shijiazhuang Tiedao University, Shijiazhuang 050043, China; 3 ProRail, 3511EP Utrecht, The Netherlands; 4 Institute of Urban Safety and Environment Science, Beijing Academy of Science and Technology, Beijing 100054, China

**Keywords:** track geometry, track degradation prediction, machine learning, Artificial Neural Network (ANN), Support Vector Machine (SVM), Grey Model (GM)

## Abstract

Keeping railway tracks in good operational condition is one of the most important tasks for railway owners. As a result, railway companies have to conduct track inspections periodically, which is costly and time-consuming. Due to the rapid development in computer science, many prediction models using machine learning methods have been developed. It is possible to discover the degradation pattern and develop accurate prediction models. The paper reviews the existing prediction methods for railway track degradation, including traditional methods and prediction methods based on machine learning methods, including probabilistic methods, Artificial Neural Network (ANN), Support Vector Machine (SVM), and Grey Model (GM). The advantages, shortage, and applicability of methods are discussed, and recommendations for further research are provided.

## 1. Introduction

**Figure 1:**
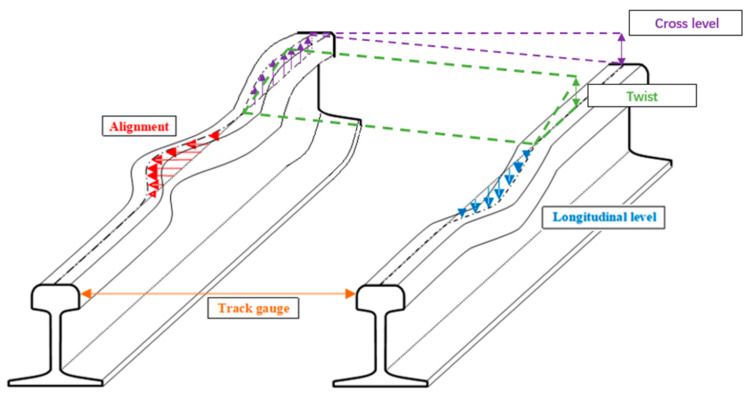
Schematic diagram of track geometry parameters.

Keeping railway tracks in good operational condition is one of the most important tasks for railway owners [1]. However, the track quality is commonly determined by various complicated factors and thus cannot be accurately predicted by mechanical algorithms or empirical methods. Instead, the track quality has to be maintained actively based on regular track geometry inspection [2]. Track geometry is the geometry of track layouts, indicating the dimensions and relative positions of each part of the railway track [3]. It typically includes gauge, twist, longitudinal level, alignment, and cross-level (also named superelevation or cant), as shown in Figure 1. The definitions of the parameters are shown in Table 1. All parameters are regularly measured at a network level to ensure the values are within a safe range for train operation [4].

**Figure 2:**
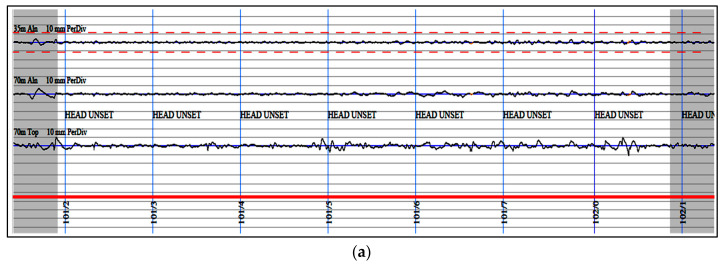

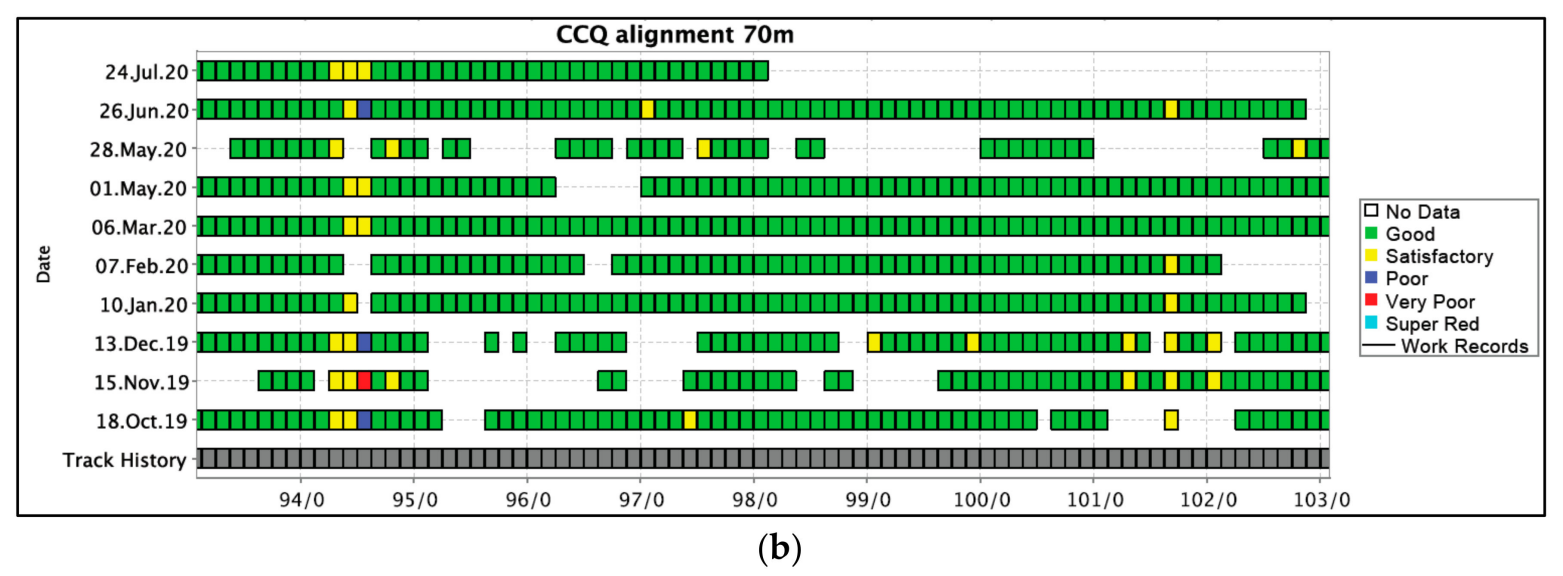
Track geometry results: (**a**) Peak value; (**b**) Average value.

Excessive deviation of track geometry can cause irregularities, affecting operation safety, train speed, and passenger comfort [10]. Therefore, it is common to access the local values and the average of a certain track length (e.g., 200 m). Peak values indicate specific locations with large irregularities (see Figure 2a, the track geometry trace of Network Rail), which is used as an evaluation index to determine the locations that need temporary maintenance, emergency repair, or speed limitation. Conversely, average values show the smoothness of a piece of track (see Figure 2b, the Colour Coded Quality Chart of Network Rail), indicating the quality of a larger range of tracks and consequently advising the track maintenance scheme at a network level [11].

To better quantify the status of track geometry, some indices have been proposed, e.g., the Track Quality Index (TQI) and Track Geometry Index (TGI) [12]. The indices are commonly based on the sum of the standard deviation of multiple track geometric parameters. Later, other parameters are integrated into the indices, e.g., track type [13], rail inclination [14], and track structure [15,16].

The common method of evaluating the track geometry condition is automated inspection using Track Recording Cars (TRCs) or Comprehensive Inspection Trains (CITs) [17]. Both contain a track geometry measurement unit, using either the initial or chord measurement methods to collect track geometry data at high speed (e.g., from 100 km/h to 300 km/h). However, because the acquisition and operation costs of TRCs or CITs are high, track geometry’s measurement frequency is limited, ranging from once per month to twice yearly worldwide. After data acquisition, the area with a large peak and average values in the track geometry is detected, and corresponding actions can be taken. Because the procedure is reactive, the track geometry inspection has to be conducted on the whole network, which is costly and time-consuming [18].

With the fast development of computer science, machine learning methods have been gradually used in the engineering industry, which provides the possibility of proactive inspection, namely extracting features from existing track geometry measurements and predictive maintenance. This can improve availability, reliability, punctuality, and safety while supporting a cost leadership strategy and improving the return of experience, paving the way for future innovations in the field of maintenance [19]. Thus, the following track inspection can only be performed at limited locations to verify the prediction results instead of at a network level [20]. More importantly, the whole track maintenance plan can be optimised. For instance, based on the prediction of track geometry, track defects can be removed in the early stage to prevent emergency maintenance in the later stage. Moreover, the track sections that need to be immediately maintained can be combined with those that will be maintained shortly to save on the maintenance cost. In this way, preventive and conditional maintenance can be achieved.

Because many machine learning models have been proposed in recent years, some machine learning-based prediction models, including the probabilistic model, Artificial Neural Network (ANN), Support Vector Machine (SVM), and Grey Model (GM), have been reviewed by Soleimanmeigouni et al. [21] in 2018, Falamarzi et al. [22] in 2019, Xie et al. [23] in 2020, and Davari et al. [24] in 2021. However, the pros and cons of some types of prediction models are not deeply discussed. For instance, the GM models that are widely used in China (written in Chinese) have not been reviewed thoroughly. Moreover, the selection of input parameters (e.g., train load, train speed, geographic location, environmental factors) and setting parameters (e.g., number of hidden layers and neurons in ANN models) of prediction models has not been reviewed. In addition, prediction models using a combination of methods have not been fully reviewed, e.g., the Principal Component Analysis-SVM model, the Grey–Markov model, and the Incremental Support Vector Regression-Bayesian model. Therefore, there is a lack of a comprehensive review of existing machine learning-based prediction models. In addition, although many researchers have used prediction models based on machine learning methods for track degradation prediction, they are not widely accepted by railway companies and are rarely used to plan maintenance activities. This is because the existing prediction models still have some limitations. For example, the current machine learning-based track degradation prediction models, such as ANN and SVM, have high complexity and are consequently difficult to interpret and the hyperparameters used in the current track degradation prediction models are determined by researchers using trial-and-error, which is time-consuming. For the above limitations, there are currently some recommendations related to future research missing.

Therefore, this paper aims to provide a comprehensive review of existing machine learning-based prediction models, with a focus on the pros and cons of prediction models, parameter selection, and models using a combination of methods. The paper can propose suggestions for future research to improve the existing machine learning-based prediction models and provide guidance and suggestions for railway companies to apply machine learning-based prediction models for better planning maintenance activities. The paper is organised as follows. First, the traditional prediction methods (non-machine learning-based prediction models) are briefly introduced in Section 2. Then, the existing machine learning-based prediction methods are introduced in detail in Section 3, including probabilistic, ANN, SVM, GM, and combination models. Section 4 discusses the prediction models’ pros and cons, and recommendations for further research are provided. Finally, the conclusions are summarised.

## 2. Traditional Prediction Methods

Traditional track degradation prediction methods are based on empirical formulas or statistical analysis [25]. They can clarify the relationship between influence factors and track degradation but cannot consider uncertainties, i.e., the heterogeneity of track sections. However, when sufficient track geometry data measured by TRCs or CITs is provided, uncertainties can be considered by the prediction methods based on the statistical analysis [26].

### 2.1. Prediction Models Based on Empirical Formulas

There have been many prediction models based on empirical formulas proposed. In this section, only some typical prediction models are introduced. More detailed reviews can be found in Guler et al. [27] and Dahlberg [28].

Sato [29] has proposed a formula for track degradation which considers the effects of track settlement, train load, speed, and track structure, shown as follows.

(1) $$S=2.09\times 10^{-3}\times T^{0.31}\times V^{0.98}\times M^{1.10}\times L^{0.21}\times P^{0.26}$$

Where *S* is the track settlement (mm/100 days); *T* is the passed tonnage (million tons/year); *V* is the average running speed (km/h); *M* is the structure factor; *L* is the effect factor for jointed rail or CWR (1 for CWR and 10 for jointed); *P* is the effect factor for subgrade (1 for good and 10 for bad).

Gao and Zhai [30] have proposed more developed prediction models considering the coupling between track responses and vehicle dynamics. Using the model, the effect of the track with initial subsidence can be considered, and the cumulation of track subsidence under repeated track load can be calculated more accurately. 

**Figure 3:**
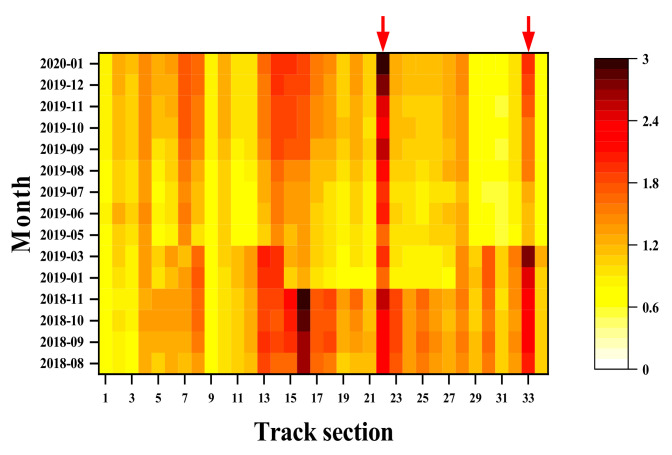
Memory effect of track degradation.

Shenton [31] has combined laboratory and field tests to study the mechanisms of track degradation. It has been found that ballast settlement plays the most important role in track degradation, and the track has a ‘quality inheritance’ or ‘memory effect’, as shown in Figure 3. After multiple maintenance cycles, the track with an excellent initial condition is likely still in good condition, while the track with a poor initial condition likely deteriorates.

Compared to other components, the ballast is easily deteriorated by train loads, external pollution, and maintenance activities [32,33]. For instance, tamping can damage ballast particles and reduce the lifetime of ballast. Thus, prediction models focused on ballast settlement have been proposed, e.g., in [34,35,36]. Sadeghi et al. [37] consider the influences of fouling and contamination on ballasts and have developed a prediction model for ballast settlement, after conducting a series of cyclic ballast box tests, shown as follows.

(2) S=α×ln(N)+β

where *S* and *N* denote the ballast settlement in (mm) and the number of loading cycles, respectively. *α* and *β* are empirical parameters that are functions of the percentage of contamination.

It should be noted that the applicability of empirical formulas is discussable. An experimental study performed by the Transportation Test Center (TTC), the American Association of Railroads (AAR), and British Railways showed that the measurement results in the United States and the United Kingdom were quite different, and few commonalities have been found [38]. Moreover, empirical formulas are limited when considering various uncertain factors related to the track, vehicle, and environment. Ignoring one factor can greatly diverge from reality [21]. Sometimes, the track sections under very similar conditions can show totally different degradation behaviours [39].

### 2.2. Prediction Models Based on Statistical Analysis

Some typical prediction models based on statistical analysis are briefly introduced in this section. A more detailed review can be found in [21].

#### 2.2.1. Linear, Multi-Stage Linear, and Exponential Prediction Models

According to Jovanovic et al. [39], track degradation has approximately linear characteristics in short time intervals. Thus, a linear degradation model can predict the track condition during a short maintenance period. Following this idea, predicting the TQI has been implemented in the railway industry, e.g., in the PWMIS (Public Works Management Information System) [40]. Xu et al. [41] have proposed using a linear regression equation to predict the short-term track irregularity for the unit track section. Jia et al. [42] have proposed a similar short-term prediction model using the linear Autoregressive Moving Average method (ARMA). 

Some researchers have used the multilevel linear regression method to predict the nonlinear behaviour of track degradation between two consecutive maintenance actions for a more extended period. For example, Chang et al. [43] have proposed a multistage linear model to predict the change of track irregularity in different phases. Similarly, Guo and Han [44] have used multistage linear models to predict various phases of track degradation between two consecutive maintenance interventions. 

Some researchers have used exponential functions to develop prediction models that can achieve higher accuracy, e.g., the prediction model in Zhou [45]. Akihito et al. [46] have proposed an exponential smoothing method to predict the standard deviation of alignment after 180 days and found that the prediction error is improved by 40% compared to the linear regression model. It should be noted that the section length in prediction models varies much, e.g., 5 m in [47] and 200 m in [48].

#### 2.2.2. Prediction Models Based on Stochastic Processes

The prediction models based on stochastic processes used in railways include the Wiener process, the Gamma process, and the Inverse Gaussian process. Meier-Hirmer et al. [49] have used the Gamma process to predict the changes in the standard deviation of track longitudinal level and found that the Gamma process is suitable for modelling the cumulative damage in tracks. Later, Meier-Hirmer et al. [50] applied a bivariate Gamma process to predict track degradation, which considers two track geometry parameters, including longitudinal level and cross level. It has been found that the Gamma process is inaccurate when the track degradation process is not monotonic, and thus the Wiener process is recommended. Zhu et al. [51] have used the Gaussian process to predict track longitudinal level and alignment. It should be noted that prediction models based on stochastic processes can be inaccurate when the variance terms in the model are significantly more extensive than the model’s mean term [5]. 

Compared to prediction models based on empirical formulas, prediction models based on statistical methods consider the local effect by using inspection data and improve prediction accuracy. However, they have the following shortcomings:

1. Models need a large sample of inspection data to reach an acceptable level of accuracy.
2. Models are barely updated with new inspection data.
3. Models lack a mechanical understanding of the relationship between factors, sometimes resulting in some unrealistic results [7].

Due to the above limitations of traditional methods, prediction methods using machine learning methods have been proposed in the railway industry, which can better reflect the random characteristics of the track degradation and even find unknown factors through data mining and eventually deliver more accurate prediction results.

## 3. Prediction Methods Based on Machine Learning

Machine learning algorithms can improve performance by self-learning, which can develop a model based on sample data (known as training data) to make predictions or decisions without being explicitly formulaic. As a result, prediction methods using machine learning algorithms are more accurate in fields where it is difficult or unfeasible to develop conventional algorithms, such as areas with many unpredictable factors. Because data acquisition has become increasingly accessible, railway engineering has entered the big data era, and thus, machine learning prediction methods have become popular. Table 2 summarises some of the recent machine learning applications in railway engineering, and the probabilistic methods ANN, SVM, and GM are the most common machine learning methods used to predict track degradation in railways.

### 3.1. Prediction Models Based on Probabilistic Method

The most important task of prediction methods based on machine learning is to estimate unknown variables (such as labelling) and make predictions. The probabilistic model can provide a description framework that converts machine learning tasks into calculating the probability distributions of input and output variables. More specifically, the probabilistic model can first calculate a stochastic matrix by analysing observable variables and inferring the stochastic matrix for all variables. It can be used to predict track degradation because track degradation has randomness and is affected by many factors, including train loads, transportation conditions, materials, and weather conditions. 

In probabilistic models for railways, the variables can be determined separately in each track section according to influencing factors, e.g., 50 m or 200 m. The prediction can be conducted in every track section and thus more accurately. Probabilistic models can adopt the Markov, Bayesian, Monte Carlo, and Particle Filter algorithms. Among them, the Markov models and Bayesian models are more commonly used.

#### 3.1.1. Prediction Models Based on Markov Theory

The Markov model is an effective tool for predicting the degradation of structures [63], which considers structure degradation a discrete process according to conditions. The transition from one condition to another in discrete time intervals is random and characterised by a probability [64]. It has been used to predict accumulative damage for various engineering structures, such as bridges and infrastructures [65,66]. Markov models have been used to predict track degradation, wherein the transition probability reflects the relationship between random factors and track degradation. The input data (track inspection data) is assumed to follow the probability distribution and is used to calculate the characteristic matrix (called a stochastic matrix or Markov matrix) to predict track geometric parameters. Markov models are suitable for data with large random fluctuations, e.g., track degradation between two consecutive maintenance interventions. It has been used to analyse the track degradation rate after maintenance in the British railway network [67] and to predict track degradation in tram tracks in Melbourne [26].

Shafahi and Hakhamaneshi [68] have used the Markov model to predict track degradation. They divide the track into six classes according to train loads, geographical location, and track conditions, which are described by five categories using a Combined Track Record index (CTR). The results show that the Markov model is better than conventional regression models, such as the ORE model. A similar conclusion has been found by Shafahi et al. [69]. They have used four models to predict track degradation: the ORE model, Markov model, ANN model, and Fuzzy Neural Network (FNN) model. The results of the Markov model are the most accurate, as shown in Table 3. Lyngby et al. [70] have proposed a modified Markov model to calculate track degradation wherein the effect of track layout (straight, curved, or a transition section) on the degradation rate is considered. Bai et al. [71] have proposed a Markov model to evaluate track degradation between two consecutive maintenance interventions. They have found that some heterogeneous factors can explain the difference in tract degradation rates in tracks (such as accumulated gross tonnage and train speeds), which should be considered in the stochastic matrix.

#### 3.1.2. Prediction Models Based on Bayesian Theory

Like the Markov model, the Bayesian model uses previous finite states to predict the next state. The difference is that the Bayesian model is more flexible than the Markov model. The relationship between states is calculated more accurately in the Bayesian model by a probabilistic mechanism that learns from data. Thus, Bayesian models have also been used to predict track degradation. 

Andrade et al. [72] have used Bayesian models to evaluate track degradation. The uncertainty of the initial standard deviation of the longitudinal level measured after renewal or tamping operations and the degradation rate in the degradation process is considered. Tanaka et al. [73] have used a similar method, wherein the position error in historical track irregularity data is first corrected to improve the prediction accuracy. Andrade et al. [74] and Yang [75] also used Bayesian models to predict track degradation. The models use the standard deviation of longitudinal level and the standard deviation of alignment as indicators. A conditional autoregressive term is added to consider the spatial interaction of adjacent track sections and avoid the prior distribution’s effect on the posterior estimation. The results show that the Bayesian model can reduce the uncertainty of track degradation and consider tamping and renewal’s effect on track degradation. Jamshidi et al. [76] have developed a Bayesian-based model for assessing rail failure (including optimistic, average, and pessimistic scenarios) probability in railway infrastructure. The results show that the model is effective for evaluating rail failure risk. In the above models, the prior available data and experience in the railway system for making maintenance decisions are not taken into account. Therefore, Movaghar and Mohammadzadeh [77] have proposed a novel method to consider the inherent uncertainties in the railway track degradation model using data elicited from expert prior beliefs in the Bayesian framework.

#### 3.1.3. Prediction Models Based on Monte Carlo Algorithm

Compared with Markov and Bayesian models, the Monte Carlo algorithm can directly solve problems with statistical properties. It is not necessary to consider structural degradation as a discrete process. The Monte Carlo algorithm generates sampling results to calculate parameters when the problem has probabilistic characteristics. As the number of simulations increases, it can get stable results by averaging the estimation of various parameters. Quiroga et al. [78] have proposed the Monte Carlo method to obtain solutions for track geometry degradation, which can be used to assess the effect of the maintenance activities. 

A sequential Monte Carlo algorithm is the Particle Filter algorithm, a state estimation method for nonlinear and non-Gaussian problems. The algorithm represents the posterior probabilities with a set of weighted random samples in a time-dependent system. Mishra et al. [79] have proposed using the Particle Filtering algorithm to model railway track degradation. The prediction results based on the particle filter are better than the results of the regression method. The main advantage of the Particle Filtering model is that it can generate probability results according to uncertain input parameters. However, since the Monte Carlo algorithm usually requires more calculation steps, it is seldom used independently to predict track degradation. It is generally used to form a Markov Chain-Monte Carlo simulation for the complex calculation of the Bayesian model in [74].

### 3.2. Prediction Models Based on ANN

**Figure 4:**
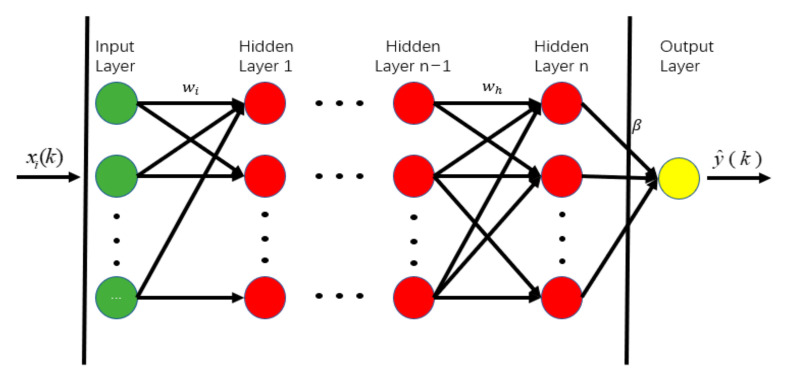
Structure of ANN model.

The ANN is a mathematical model that simulates the connection structure of human brain neurons for information processing. The ANN model can be divided into input, output, and hidden layers (see Figure 4). Each node in the input layer corresponds to a predictor variable, and the node in the output layer corresponds to the target variable. Between the input layer and the output layer are hidden layers. The number of hidden layers and the number of nodes (neurons) in each layer determine the complexity of the ANN model [10]. ANN models with multiple non-linear hidden layers can learn complicated relationships between input and output variables. However, limited training data may contain the results of sampling noise, resulting in a complex relationship between input and output variables. This complex relationship may not exist in actual test data, leading to overfitting [80].

Other neural networks are the Convolutional Neural Network (CNN), Recurrent Neural Networks (RNN), and Deep Neural Network (DNN). The CNN is mainly designed for image data [81,82,83], and the RNN is widely used to analyse time series and sequence data [23,84]. The DNN can be used for track component classification [85], railway defect detection [86], and track settlement prediction [87]. The ANN is commonly used for engineering to explore the importance of input features to output parameters. Moreover, the ANN model is relatively simple compared to other techniques [6]. Therefore, this section mainly introduces the application of ANN models in track degradation prediction.

The hidden layers of the ANN model improve the network’s mapping accuracy. The number of hidden layers is proportional to the complexity of the research object. Experiments usually determine the number of hidden layers for track degradation prediction. Sadeghi et al. [88] have developed an ANN model with a single hidden layer to predict structural defects in railway tracks. The model considers the standard deviation of track geometry data as inputs and component defects (defects in rail, sleeper, ballast, and fasteners) as outputs. It has been found that the prediction accuracy varies with the combinations of inputs and neurons in the hidden layer, as shown in Table 4. Peng et al. [89] have used ANN models to predict track geometry irregularity and found that the ANN model with two hidden layers is better than with a single hidden layer. Falamarzi et al. [90] have used ANN models with two hidden layers to predict the track gauge of tram lines under different maintenance conditions (repaired or unrepaired) and track layouts (curve or straight). It has also been found that the combination of neuron numbers in hidden layers affects prediction accuracy. For instance, the ANN model with 15 neurons in the first hidden layer and 10 in the second obtains the best prediction accuracy for repaired straight tracks; the ANN model with 20 and 15 neurons in hidden layers is optimal for the prediction of repaired curve tracks; and the ANN model with 25 and 20 neurons is optimal for the prediction of unrepaired straight and curve tracks.

When using the ANN model for predictions for railway tracks, the choice of input variables strongly impacts the prediction accuracy. When too many input variables are used, the calculation efficiency of the ANN model is low; when too few input variables are used, the prediction accuracy of the ANN model is low. In practice, input variables are commonly selected based on personal experience. After that, the correlation between each input variable and the prediction is analysed. The input variables with higher correlation and less independent of each other are selected to improve the efficiency and accuracy of prediction models. For instance, Shafahi et al. [69] used an ANN model to predict track degradation, which considered six input variables, including the CTR index, train load, train speed, geographic location, and the gradient of the track section, and the track layout. The ANN model developed by Guler et al. [91] considers more input variables, including cross level, rail and sleeper type, and environmental factors (falling rock, landslide, snow, and flood). However, the authors in Lee et al. [92] think the environmental factors are less important and consider the subgrade type and maintenance parameters (the number of compactions on ballast and initial TQI) instead. In the work of Khajehei et al. [93], the authors used the Garson algorithm to calculate the relative importance of input variables, as shown in Table 5, and found that the maintenance record, track degradation rate after tamping, and train load are more relevant to prediction accuracy than other variables. 

A summary of the input and output variables considered in existing studies is shown in Table 6.

### 3.3. Prediction Models Based on SVM

**Figure 5:**
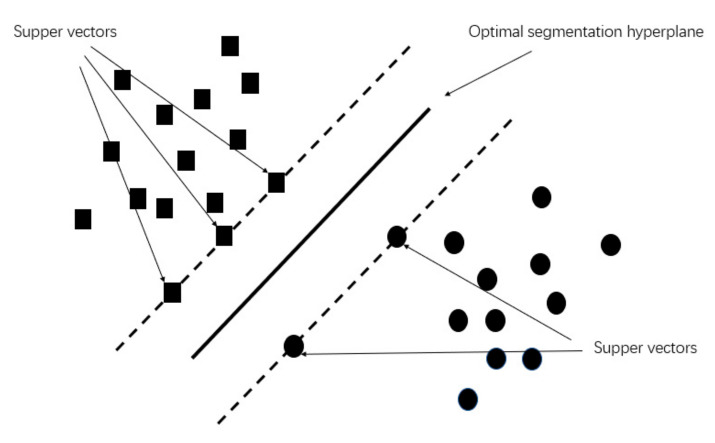
Schematic diagram of SVM.

The SVM (Support Vector Machine) is a popular machine learning model for classification and regression prediction [94]. The SVM can generally be divided into two categories: Support Vector Classification (SVC) and Support Vector Regression (SVR). The SVM can assign new examples to one category or another by transforming the input space into a high-dimensional space using an optimal classification surface, as shown in Figure 5. Four kernel functions are commonly used to find optimal classification surfaces, including linear, polynomial, radial, and sigmoidal functions [95]. The SVM is good at solving data with a small sample, nonlinearity, and high dimensions and thus has been used for pattern recognition, regression analysis, and time series prediction for track degradation.

The prediction accuracy of the SVM model is also strongly determined by input variables. Considering train load, speed, track layout, track class, time intervals, and length and amplitude of defects, Hu et al. [96] have used the SVM model to predict the track geometry defects, including cross level, longitudinal level, and twist. The results show that the SVM model achieves more than 70% prediction accuracy for track geometry defects. Falamarzi et al. [97] have used the Pearson correlation analysis to analyse continuous input variables, including train load, track layout, and gauge, and the One-way Analysis of Variance (ANOVA) to analyse categorical input variables, including rail support, rail profile, surface, and rail type. The statistical analyses show that the gauge and rail type are significant for predicting the gauge in straight sections, while the rail type is insignificant for predicting the gauge in curved sections. They have compared the prediction results (gauge) of the SVM model and that of the ANN model and found that the prediction accuracy of the SVM is higher in curved sections while lower in straight sections, as shown in Table 7. Bergmeir et al. [98] have used the SVM model to predict incidence on railway tracks, which considers the acceleration of the bogie, axle box, and car body as input and compared the prediction results to other prediction models, including neural networks and statistical autoregression models. The results show that the Root Mean Squared Forecaster Error (RMSFE) of the SVM model is 0.7, which is more suitable for incidence prediction than others. 

**Figure 6:**
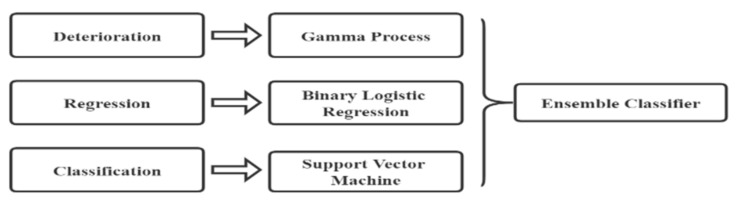
Structure of ensemble classifiers. Adapted from [99].

The prediction accuracy of the SVM model can be improved when combined with another algorithm. Gallo et al. [99] have proposed two ensemble classifiers (aggregation and stacking) to predict cross level, longitudinal level, and twist. The classifiers consider three perspectives: degradation, regression, and classification, modelled by the gamma process, binary logistic regression, and SVM separately, as shown in Figure 6. The results show that the accuracy of the ensemble methods for predicting longitudinal level and twist is higher than that of the single SVM model, while it is lower for predicting cross level, wherein the detailed improvements are shown in Table 8.

Lee et al. [100] have developed the Online Support Vector Regression (OSVR) model to predict the TQI, which is a regularly updated SVM model without retraining all historical measurement data. The results show that the prediction accuracy of the OSVR model is better than that of SVR by about 10%, while the OSVR model is unsuitable for predicting incremental data sets. After that, they combined an SVM model (Incremental Support Vector Regression, referred to as ISVR) with Bayesian optimisation. The results show the prediction accuracy of the combined model is better than that of SVR by about 20% because Bayesian optimisation can realise the automatic tuning of hyperparameters in the ISVR model. 

Lin et al. [101] introduced Principal Component Analysis (PCA) to the SVM model and developed a PCA-SVM prediction model for the track geometry defects. The PCA is used to extract the main elements of the axle box acceleration characteristic parameters, which are considered as input to the SVM model. The results show that the PCA-SVM model can achieve 75.5% prediction accuracy for track geometry defects. Xu et al. [102] have proposed the SVM-Monte Carlo (SVM-MC) method to construct a stochastic prediction model for the track longitudinal level. The SVM model is used to calculate the parameters of the stochastic prediction model, which are used as input for the Monte Carlo method to predict the track longitudinal level. The results show that the Mean Relative Error (MRE) of prediction results of the SVM-MC model is 4.63%, which can provide technical support for maintenance plan decisions. A summary of the input and output variables considered in existing studies is shown in Table 9. 

### 3.4. Prediction Models Based on GM

Deng [103] has proposed the grey system theory, which mainly studies the uncertainty caused by small and insufficient samples. The grey system explores the potential pattern of research objects by screening and extracting parts of known information [104]. The grey prediction theory is an important part of the grey system theory, which can be used to predict the future change of a research object. In the grey prediction theory, the GM (n, m) represents a grey model, where n is the order of the difference equation and m is the number of variables. The GM (1, 1) model is widely used due to its high computational efficiency [105]. Because track degradation is complex and the correlation between factors is uncertain, prediction models based on the GM (1, 1) have been developed. 

When using GM (1, 1) models to predict variables with time series, e.g., TQI, they can be divided into equal and unequal time interval models. The GM (1, 1) with unequal time intervals is commonly used when track maintenance is considered. Using the GM (1, 1) model to predict the track degradation mainly focuses on the optimisation and extension of the GM (1, 1). Qu et al. [106] have developed an improved GM (1, 1) model to predict the TQI. They use the integration method to reconstruct the GM (1, 1) and introduce a periodic function to correct the residual of TQI. The results show that the MRE of prediction results of this model is 0.88%, which indicates this model has high prediction accuracy. Later, Qu et al. [107] introduced time-varying parameters to the GM (1, 1) model in [100] to predict the TQI. The results show that the MRE of prediction results for this model is 0.69%, which is lower than that of the GM (1, 1) model in [106]. 

The traditional GM (1, 1) is only good at predicting the sequence with an exponential increase. Jia [108] has developed a linear recursive GM (1, 1) model to predict the standard deviation of longitudinal level and cross level by improving data preprocessing, background value reconstruction, and model deviation processing. Similarly, Xu [109] has proposed a recursive liner GM (1, 1) model with residual modification for the prediction of TQI. These models all introduce the correction function to correct the residual sequence of the TQI. Guo et al. [110] have developed a GM (1, 1) model with an updated mechanism to predict track degradation. The results show that the MRE of prediction results of the unit track section (200 m) is 1.95%, and that of the continuous track sections (600 m) is 3.62%, which indicates that the model has good prediction accuracy for track degradation of different sections. Wang et al. [111] and Bao et al. [112] both proposed a grey interval prediction model based on the GM (1, 1) to predict the track degradation of passenger railway lines and heavy haul railway lines, respectively. The MRE of prediction results is 0.07% and less than 0.05%, respectively, which shows grey interval prediction models can be used for different types of railway lines. 

With the rapid development of monitoring technology, the amount of inspection data has increased, and the random fluctuation of data occurs more often. It becomes more challenging to predict track degradation accurately from big data with significant randomness using single GM (1, 1) models. Thus, GM (1, 1) models have also been combined with other algorithms for better prediction accuracy. 

According to Qu [113], the development of TQI is composed of deteriorating trend components and random fluctuation components. Because Markov models can better predict the random fluctuation components, the Grey–Markov model has been developed for track degradation prediction, which can compensate for the drawbacks of the single GM (1, 1) model and achieve higher prediction accuracy [114]. Liu et al. [115] have combined the GM (1, 1) model with the centre approach (introduced in [116]) to combine with the Markov model to predict TQI. The results show that the model has better prediction accuracy than that of a single GM (1, 1). Pan et al. [117] have compared the single GM (1, 1), Gray–Markov, and linear regression models and found that the Grey–Markov model has the best prediction accuracy for TQI with strong randomness in a long time.

Because the neural network models can predict problems with complex nonlinearity or uncertainty problems, the combination of the neural network model and the GM (1, 1) model has been studied. Han et al. [118] have combined the Back Propagation (BP) Neural Network model with the GM (1, 1) to form the BP-GM model to predict TQI. The BP Neural Network model is used to correct the residual values of the TQI predicted by the GM (1, 1) model. Compared with the TITCGM (1, 1)-PC model (introduced in [113]), the MRE of prediction results of the BP-GM model decreased from 4.59% to 2.42%. However, the prediction results of the BP-GM model are unstable because the initial weights and thresholds of the BP Neural Network are randomly assigned. To solve this problem, Tang [119] has optimised the BP Neural Network using the Mind Evolutionary Algorithm (MEA) and developed a GM-MEA-BP model. Thanks to the global optimisation ability of MEA (introduced in [120]), the convergence speed of the BP Neural Network is improved, and the mistake of the BP Neural Network falling into local optimum is solved. Ma et al. [121] have combined the GM (1, 1) with the Elman Neural Network optimised by a Genetic Algorithm (GA) to develop the GM-GA-Elman model to predict TQI, wherein the optimised Elman Neural Network is used to correct the residual values of TQI. Compared with the TITCGM (1, 1)-PC model (introduced in [113]) and the GM-BP model (introduced in [118]), the MRE of prediction results of the GM-GA-Elman model decreased from 5.74% and 2.59%, respectively, to 1.89%. Yao [122] has developed a prediction model (GM-RNN) combined with the GM (1, 1) model and RNN. The GM-RNN model considers the characteristics of the time series of TQI to improve prediction accuracy. Compared with the GM-BP model (introduced in [112]) and the GM-GA-Elman model (introduced in [121]), the MRE of prediction results of the GM-RNN model decreased from 3.12% and 1.88%, respectively, to 1.52%. 

**Figure 7:**
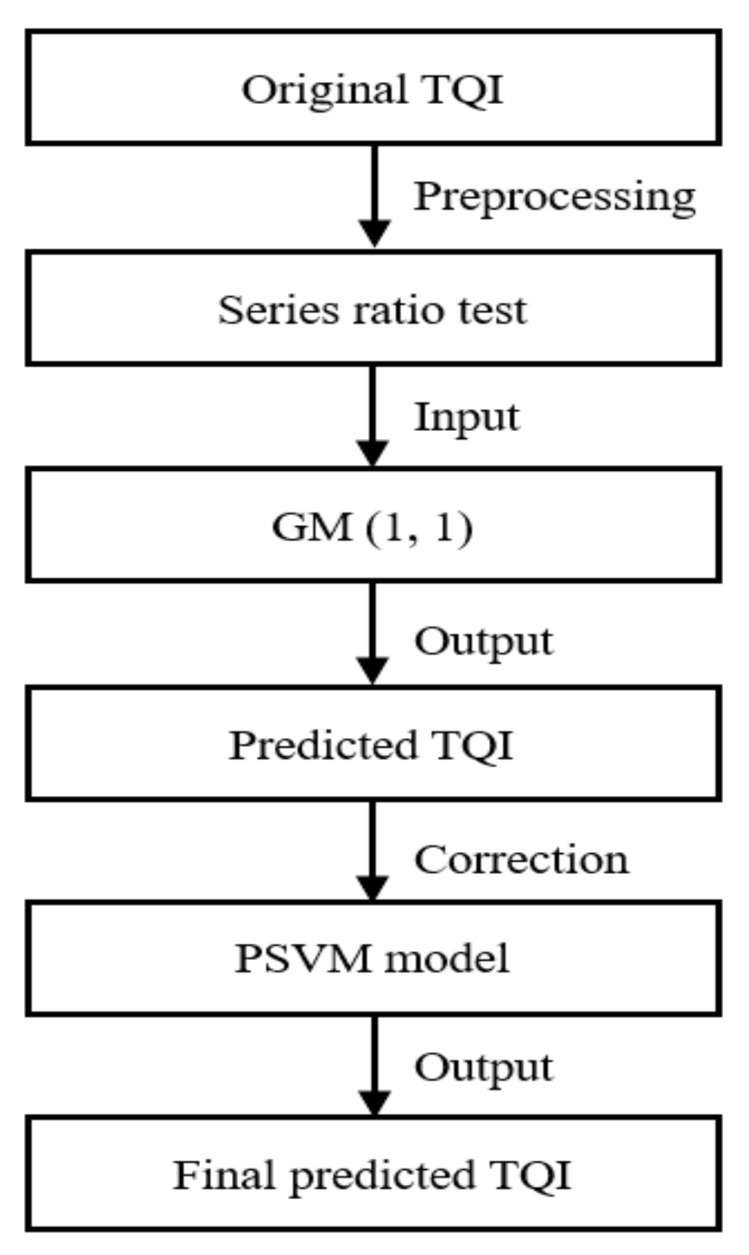
Structure of GM-PSVM model.

The SVM models can better solve data with a small sample, nonlinearity, and high dimensions. The SVM models have also been combined with GM models to improve prediction accuracy. Ma et al. [123] have proposed a prediction model (GM-PSVM) that combined the GM (1, 1) and SVM models optimised by the Particle Swarm Optimization (PSO) (introduced in [124]). The PSO can automatically find the best parameters for SVM and effectively avoid the influence of improper parameter selection on SVM. In the GM-PSVM, the GM (1, 1) model is used to predict the TQI, and the residual values of TQI are corrected by the PSVM model, as shown in Figure 7. Compared with the TITCGM (1, 1)-PC model (introduced in [113]) and the GM-BP model (introduced in [118]), the MRE of prediction results of the GM-PSVM model decreased from 5.07% and 3.48%, respectively, to 2.74%. Feng et al. [125] have proposed a prediction model (GM-WOA-LSSVM) to predict TQI, which combines the GM (1,1) model and the Least Squares SVM (LSSVM) model (introduced in [126]) and is optimised by the Whale Optimization Algorithm (WOA) (introduced in [127]). The WOA can also automatically find the best parameters for SVM. Compared with the GM-BP model (introduced in [118]), the MRE of prediction results of the GM-WOA-LSSVM model decreased from 2.29% to 1.99%.

Some researchers have also adopted other methods to improve the prediction accuracy of GM (1,1) models. Jia et al. [42] have shown that the GM (1, 1) model can predict the cross level at fixed measuring points in the long term after residual modification. In addition, they used the Autoregressive, Kalman Filter, and ANN models for the short-term prediction of the cross level at the unit track section. The results show that combining these four models can predict the cross level at fixed measuring points and unit track sections in the short and long term. Li [128] has developed an ESGM-RGCD model to predict TQI, which combines the Exponential Smoothing model and Relative Grey Correlative Degree (introduced in [129]) with the GM (1, 1) model. The results show that the prediction accuracy of the ESGM-RGCD model is better than that of the Exponential Smoothing model and GM (1, 1). Xin et al. [130] have combined the GM (1, 1) with the Fourier Series (introduced in [131]) to develop the FGM prediction model for TQI, wherein the Fourier Series is used to correct the residual value of TQI predicted by the GM (1, 1) model. The results show that the FGM model presents the best performance compared with the linear model, exponential model, GM (1, 1), and Grey–Markov model.

The existing predictive models combing the GM (1, 1) model and other models are shown in Table 10.

## 4. Discussions and Perspectives

**Figure 8:**
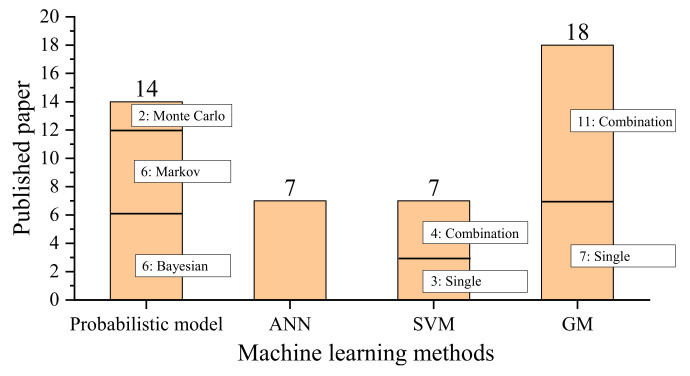
Statistical analysis for existing machine learning methods for track degradation prediction.

There are 46 studies reviewed in Section 3, and the machine learning methods are shown in Figure 8. The most commonly used machine learning method is GM (39%), followed by the probabilistic model (30%), ANN (15%), and SVM (15%). For probabilistic models, the most commonly used models are the Markov model (43%) and the Bayesian model (43%). Moreover, the GM and SVM methods are mostly combined with other methods, which shows that combined models have become increasingly popular.

### 4.1. Pros and Cons of Prediction Models

Track degradation is determined by many factors, including train load, track layout, speed, rail type, weather conditions, etc. Even if two track sections have similar conditions, the degradation rate may still differ. Probabilistic models can consider this difference in predicting track degradation. The track condition can be predicted separately in each section, e.g., 50 m or 200 m, and thus more accurately. However, it is worth noting that probabilistic models also have some limitations.

- There are three limitations in Markov models: (1) The model is discrete while the track geometric is continuous, which may lead to inaccurate prediction. (2) Defining a large number of discrete states for track degradation leads to a complex calculation using a stochastic matrix. (3) When using different stochastic matrices for track sections, the complexity of Markov models is even further increased.
- The limitation of the Bayesian model is that the prior probability distributions of fitting track degradation parameters are assumed to be independent, while the prior probability distributions are not completely independent in practice. This assumption may lead to lower prediction accuracy.
- Monte Carlo models usually require many calculation steps and are commonly used together with other models to predict track degradation.

Compared to probabilistic models, ANN models can predict track degradation without complex matrix calculations and the prior assumption that track degradation parameters are independent. The advantages of ANN models can be summarised as follows.

- ANN models can study the correlation between input and output variables and identify the key factors for track degradation, which can simplify the prediction model and improve computational efficiency.
- ANN models have a strong computational capability and can process a larger amount of track detection data, which are suitable for predicting large-batch track geometry data.
- ANN models can learn hidden relationships in data without imposing any fixed relationships on the data, which are robust to predict the track geometry data with drastic fluctuations.
- ANN models are relatively simple compared to other Neural Network models [6], such as the CNN, RNN, and DNN models, etc.

The limitations of ANN models are as follows.

- A large amount of high-quality inspection data is required for ANN models as training data. ANN models cannot work when track geometry data is insufficient.
- ANN models have high complexity and are consequently difficult to interpret. The prediction results can lack theoretical explanation and have poor generalisation performance due to extrapolation and observational biases.
- The parameters of ANN models are commonly determined by researchers using a trial-and-error way, which is time-consuming and causes the performance of ANN models to vary from person to person.
- Because ANN models have a low convergence speed and easily fall into local optimum, the theory and learning algorithm of the ANN models need to be further improved.

Compared to ANN models, SVM models can avoid falling into local optimums. In addition, empirical components are not needed in SVM models due to the theoretical basis of SVM. Other advantages of SVM models are as follows.

- SVM models can consider many factors for track degradation because the complexity of SVM is irrelevant to the dimension of input variables.
- SVM models can still achieve high prediction accuracy when the sample amount (inspection data) is limited.
- SVM models are good at predicting track geometry data with nonlinearity and high dimensions.

However, it is difficult to train a large amount of track geometry data for SVM models. The reason is that large-batch data can increase the computational complexity of the SVM models, and a large number of peaks in data can be considered as super vectors, which can mislead the maximum margin hyperplane of the SVM models (see Figure 5). In addition, it is also difficult to select appropriate kernel functions for SVM models. To overcome this shortcoming, SVM models are often combined with other methods to optimise model parameters, increase computational efficiency, and expand the scope of application. It is worth noting that there are still more combinations of SVM models and other methods to be explored in future research.

Because track degradation is a grey system which is affected by many uncertain factors, GM (1, 1) models can be used to predict track degradation. Specifically, GM (1, 1) models can study the uncertainty caused by the small-batch track geometry data and insufficient railway track information. In addition, GM (1, 1) models can easily explore the potential rules between input and output variables due to their high computational efficiency. It is worth noting that GM (1, 1) models are more suitable for short-term prediction of track degradation with slight fluctuation (see Table 10). 

Because the development of track geometry data is composed of deteriorating trend and random fluctuation components, and because random fluctuation components often cause significant inaccuracy in the GM (1, 1) model, GM (1, 1) models are usually combined with other models to correct the residual values of the sequence and eventually achieve better prediction accuracy. In some combined models, the inspection data can be directly used without training, and long-term prediction can be performed.

### 4.2. Recommendations for Future Research

Although many researchers have used prediction models based on machine learning methods for track degradation prediction, they are not widely accepted by railway companies and are rarely used to plan maintenance activities. To improve it, some recommendations for future research are as follows.

1. The current machine learning-based track degradation prediction models, such as ANN and SVM, effectively solve problems but remain rather opaque about how they actually solve them [19]. The results can lack theoretical explanation and have poor generalisation performance due to extrapolation and observational biases. Therefore, machine learning models should be combined with mechanical models or empirical models in future research. For instance, the mechanical models or empirical models can be used to check whether predicted TQI or track geometry are within the reasonable limit or proportional to other variables.
2. In the current machine learning-based track degradation prediction models, the tracks are commonly divided into sections of a fixed length, e.g., 50 m or 200 m. The track in a section is assumed to be the same. However, the track can be very different within a section. Therefore, track sections in prediction models should be divided according to features instead.
3. The performance of machine learning models is strongly determined by the values of hyperparameters. However, the hyperparameters used in the current track degradation prediction models are determined by researchers using a trial-and-error approach, which is time-consuming. Therefore, the theory of automatically determining hyperparameters should be studied.
4. In the existing studies, the proposed machine learning-based prediction models are tested on individual railway tracks. Therefore, the generalisation performance of the models is unclear. The models should be tested for railway tracks of different types in future research to improve the generalisation performance.
5. Generally speaking, it requires certain knowledge of computer science to properly use the prediction results of machine learning models, which may be challenging for some maintenance staff or inspectors. Therefore, the prediction models should be further integrated into simple applications to improve usability.
6. Other advanced machine learning methods can also be combined into track degradation prediction models for track inspection data with large-size, multi-source, high-fluctuation, and high-noise. For instance, Deep Learning has been used in other engineering fields [59,81,132], but rarely for track degradation.

## 5. Concluding Remarks

Due to the rapid development of computer science, many machine learning models for predicting track degradation have been developed, which can extract features from existing track geometry measurements and predict future development. According to the prediction results, track inspection can only be performed at limited locations and track maintenance plans can be optimised. 

The paper reviews the existing prediction methods for railway track degradation, including traditional methods and prediction methods based on machine learning methods, including probabilistic methods, Artificial Neural Network (ANN), Support Vector Machine (SVM), and Grey Model (GM). The machine learning models can improve performance by self-learning and making predictions without explicit formulas, thus often having better prediction accuracy than traditional prediction models. The main shortcomings of probabilistic models are that prior probability distributions of fitting track degradation parameters are assumed to be independent, and the calculation of the matrix is complex. The main shortcoming of the ANN and SVM models is that their selection of model parameters lacks theoretical explanation. The main shortcoming of GM models is the high data requirement.

To improve the acceptance of prediction methods based on machine learning methods in the railway industry, some recommendations for future research are provided as follows.

- Machine learning-based prediction models should be combined with mechanical models or empirical models, which can give a theoretical explanation for the prediction results of the prediction models and enhance the generalisation performance of the prediction models. For instance, the mechanical models or empirical models can be used to check whether predicted TQI or track geometry are within the reasonable limit or proportional to other variables.
- Track sections in prediction models should be divided according to features, which can fully consider the differences of track sections to achieve fine modelling. For instance, special track sections (such as turnout zones, transition zones, etc.) can be distinguished from other track sections.
- The theory of automatically determining hyperparameters should be studied, which can achieve automatic adjustment of hyperparameters for different track geometry datasets and provide excellent prediction accuracy. For instance, the Random Search algorithm built into KerasTuner (a deep learning Application Programming Interface (API) written in Python) can be used to automatically find the best hyperparameters of the SVM or ANN models for track degradation prediction.
- Prediction models should be tested for railway tracks of different types, which can improve the generalisation performance of the prediction models. For instance, the same prediction model can be used to predict the degradation of passenger and freight railway tracks, respectively, and analyse and compare the prediction results.
- Prediction models should be further integrated into simple applications, which can allow the prediction models to be widely accepted by railway companies and used to plan maintenance activities. For instance, predictive models can be integrated into software on portable mobile phones.
- Other advanced machine learning methods can be used, such as Deep Learning, which can be used to better predict the track inspection data with large-size, multi-source, high-fluctuation, and high-noise. For instance, the Deep Neural Network (DNN) model can be used to predict track degradation.

## Figures and Tables

**Table 1:** Definition of track geometry parameters.

Track Geometry	Definition
Gauge	The distance between the inner sides of the left and right rail heads is perpendicular to the track centre.
Twist	The measurement of elevation difference between the top surfaces of two rails [5].
Longitudinal level	The geometrical error in the vertical plane is represented by the difference (in millimetres) between a point on the top of the rail in the running plane and the ideal mean line of the longitudinal profile [6].
Alignment	The deviations in the lateral positions of the left and right rails from a mean trajectory were obtained by filtering out wavelengths longer than a given length [7,8].
Cross level	The deviation between the top surfaces of two rails at a given location [9].

**Table 2:** Recent applications of machine learning methods for railway tracks. Adapted from [52].

Reference	Application	Objective	Machine Learning Technique
[53]	Sleeper inspection	Predict rail machine vision for maintenance	Classifier fusion combined models
[54]	Rail track geometry modelling	Predict rail track geometry degradation for maintenance	Hierarchical Bayesian models, Markov Chain-Monte Carlo (MCMC)
[55]	Condition-based maintenance in railway transportation systems	Use sensor data collection to maximise remaining useful life (RUL)	Big data streaming analysis, online support vector regression models
[56]	Material classification and semantic segmentation of railway track images	Extraction of accurate information from visual track inspection images	Deep convolutional neural networks
[57]	Prediction of railway track irregularities	Identify possible underlying patterns or rules for predicting	Classification learning, tree-augmented naive Bayes
[58]	Risk assessment of rail failure	Use big data techniques to process image data for automatic squat detection	Deep convolution neural network
[59]	Rail accident data reporting	Text analysis of railway accident reports	Deep learning, recurrent neural networks (RNN), long short-term memory networks (LSTM)
[60]	Data-driven artificial track quality indices	Use the principal component analysis for feature selection and combined track quality index (TQI)	Dimension reduction random forest, support vector machines
[61]	Recent applications of big data techniques in railway	Literature review	Descriptive statistics, charts, etc.
[62]	Data-driven optimisation of railway maintenance for track geometry	Track degradation modelling	Random forests, Markov methods including MCMC, Markov decision processes (MDP)

**Table 3:** The comparisons of Markov chain, ANN, FNN, and ORE models. Adapted from [69].

Model	$R^{2}$
ORE	0.12
Markov chain	0.83
ANN	0.72
FNN	0.81

**Table 4:** Settings for ANN model in [88]. Adapted from [88].

Prediction Object	Input Variable (Standard Deviation)	Neurons
Rail defect	Gauge, longitudinal level, alignment, twist	15
Sleeper defect	Gauge, longitudinal level, alignment, twist	10
Ballast defect	Longitudinal level, alignment, twist	25
fastener defect	Gauge, longitudinal level, twist	15

**Table 5:** Relative importance of input variables. Adapted from [93].

Input Variables	Relative Importance (%)	Rank
Maintenance record	16.1	1
Track degradation rate after Tamping	11.79	2
Train load	9.51	3
Train speed	7.97	4
Ballast age	7.81	5
Sleeper age	7.78	6
Sleeper type	7.45	7
Cross level	7.29	8
Rail type	6.69	9
Bridge	6.58	10
Track layout	6.32	11

**Table 6:** Input and output variables in existing studies.

Reference	Input Variables	Output Variables
[88]	Standard deviation of gauge, longitudinal level, alignment, and twist	Defect density of rail, sleeper, ballast, and fastener
[89]	Standard deviation of gauge, longitudinal level, alignment, cross level, and twist	Standard deviation of gauge, longitudinal level, alignment, cross level, and twist
[90]	Gauge, train load, track layout, ballast type, rail type, and rail support	Gauge
[69]	CTR index, train load, track layout, train speed, geographic location, and gradient of track section	CTR index
[91]	Cross level, train load, track layout, train speed, rail type, sleeper type, the gradient of the track section, and environmental factors	Track degradation rate
[92]	TQI, train load, track layout, train speed, subgrade type, and maintenance parameters	TQI
[93]	Cross level, train load, track layout, train speed, rail type, sleeper type, sleeper age, ballast age, bridge, and maintenance parameters	Track degradation rate

**Table 7:** Results of SVM and ANN for prediction of straight and curved sections. Adapted from [97].

Model	Sections Type	R2	MSE
SVM	Straight sections	0.90	1.48
	Curved sections	0.83	2.43
ANN	Straight sections	0.95	0.80
	Curved sections	0.78	3.8

**Table 8:** Overall accuracy results of single and ensemble models. Adapted from [99].

Defect	Single Classifier	Ensemble Classifier
	SVM	Support Vector Machine Stacking (SSV)
Cross level	74.55%	73.99%
Longitudinal level	78.73%	79.50%
Twist	76.28%	82.56%

**Table 9:** Input and output variables in existing prediction modes based on SVM.

Reference	Input Variables	Output Variables
[96]	Train load, train speed, track layout, track class, time intervals, and length and amplitude of defect	Track geometry defects
[97]	Gauge and rail type	Gauge
[98]	Acceleration of the bogie, axle box, and car body	Incidence in railway tracks
[99]	Train load, track class, time intervals, and length and amplitude of defect	Track geometry defects
[100]	TQI, train load, track layout, train speed, subgrade type, and maintenance parameters	TQI
[101]	Time-domain and frequency domain of axle box acceleration	Track geometry defects
[102]	Mean and variance of the parameters of a track degradation model	Mean and variance of the parameters of a track degradation model

**Table 10:** Existing predictive models combined GM (1, 1) model and other models.

References	Model	Application	Advantage	Limitation
[113]	TITCGM (1,1)-PC	Short-term prediction for the TQI	Respectively consider the deteriorating component and random component in the TQI	Poor prediction accuracy
[114,115,117]	Grey-Markov	Long-term prediction of the TQI	Predict the TQI with strong randomness	Need to build complex transition matrixes
[118]	GM-BP	Short-term prediction for the TQI	Improve the prediction accuracy of the GM (1, 1)	Lack of prediction stability
[119]	GM-MEA-BP	Short-term prediction for the TQI	Improve the convergence speed of the BP Neural Network	The time-series properties of the TQI are not considered
[121]	GM-GA-Elman	Short-term prediction for the TQI	Avoid the BP Neural Network instability caused by random assignment	Need to study the applicability of the model in the medium and long term
[122]	GM-RNN	Short-term prediction for the TQI	Consider the time-series properties of the TQI	Lack of prediction stability
[123]	GM-PSVM	Short-term prediction for the TQI	Solve the problem of BP Neural Network, which easily falls into local optimum	Need to study the applicability of the model in the medium and long term
[125]	GM-WOA-LSSVM	Short-term prediction for the TQI	Need few optimisation parameters and improve the learning speed	Need to study the applicability of the model in the medium and long term
[42]	GM-AR	Long-term and short-term prediction for the TQI	Realise long-term and short-term prediction for the TQI	Need to consider more random factors
[128]	ESGM-RGCD	Short-term prediction for the TQI	Obtain the optimal solution of the weight coefficient of the combined model	Need to study the applicability of the model in the medium and long term
[130]	FGM	Short-term prediction for the TQI	Improve the prediction accuracy of the GM (1, 1)	Need to study the applicability of the model in the medium and long term

## Data Availability

The data presented in this review can be requested from the corresponding author or the first author.
